# *fac*-(2-Amido­ethyl-κ^2^ *C* ^1^,*O*)trichlorido(urea-κ*O*)tin(IV)

**DOI:** 10.1107/S1600536811038281

**Published:** 2011-09-30

**Authors:** R. Alan Howie, Geraldo M. de Lima, Edward R. T. Tiekink, James L. Wardell, Solange M. S. V. Wardell

**Affiliations:** a Department of Chemistry, University of Aberdeen, Meston Walk, Old Aberdeen, AB24 3UE, Scotland; b Departamento de Química, Instituto de Cie^ncias Exatas, Universidade Federal de Minas Gerais, Avenida Anto^nio Carlos, 6627 Pampulha, 31270-901 Belo Horizonte, MG, Brazil; c Department of Chemistry, University of Malaya, 50603 Kuala Lumpur, Malaysia; d Centro de Desenvolvimento Tecnológico em Saúde (CDTS), Fundação Oswaldo Cruz (FIOCRUZ), Casa Amarela, Campus de Manguinhos, Av. Brasil 4365, 21040-900, Rio de Janeiro, RJ, Brazil; e CHEMSOL, 1 Harcourt Road, Aberdeen AB15 5NY, Scotland

## Abstract

The Sn atom in the title compound, [Sn(C_3_H_6_NO)Cl_3_(CH_4_N_2_O)], is octa­hedrally coordinated within a CCl_3_NO donor set provided by a chelating amido­ethyl ligand (C and O), a urea-O atom and three facially arranged Cl atoms. Systematic variations in the Sn—Cl bond distances are correlated with the relative *trans* influence exerted by the C and carbonyl-O atoms. The three-dimensional crystal packing is stabilized by N—H⋯O and N—H⋯Cl hydrogen bonds.

## Related literature

For background and for related Sn[OCH(NH_2_)CH_2_CH_2_]Cl_3_ *L* structures, see: Howie *et al.* (2011 ▶); Wardell *et al.* (2010 ▶); Tiekink *et al.* (2006 ▶).
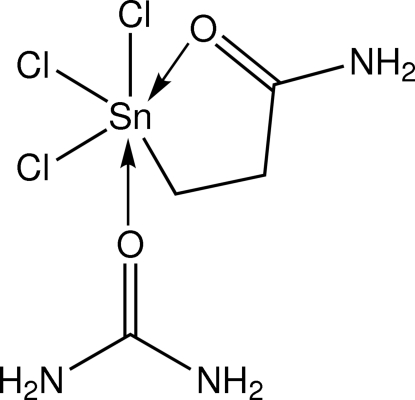


## Experimental

### 

#### Crystal data

[Sn(C_3_H_6_NO)Cl_3_(CH_4_N_2_O)]
*M* *_r_* = 357.19
Orthorhombic, 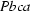

*a* = 11.1223 (2) Å
*b* = 12.0180 (3) Å
*c* = 16.0461 (5) Å
*V* = 2144.85 (9) Å^3^
*Z* = 8
Mo *K*α radiation
μ = 3.10 mm^−1^
*T* = 120 K
0.14 × 0.08 × 0.03 mm

#### Data collection

Bruker–Nonius APEXII CCD camera on κ-goniostat diffractometer
Absorption correction: multi-scan (*SADABS*; Sheldrick, 2007 ▶) *T*_min_ = 0.738, *T*_max_ = 0.913
12333 measured reflections
2451 independent reflections
2242 reflections with *I* > 2σ(*I*)
*R*_int_ = 0.039

#### Refinement

*R*[*F* ^2^ > 2σ(*F* ^2^)] = 0.022
*wR*(*F* ^2^) = 0.051
*S* = 1.10
2451 reflections
136 parameters
H atoms treated by a mixture of independent and constrained refinement
Δρ_max_ = 0.42 e Å^−3^
Δρ_min_ = −0.41 e Å^−3^

### 

Data collection: *COLLECT* (Hooft, 1998 ▶); cell refinement: *DENZO* (Otwinowski & Minor, 1997 ▶) and *COLLECT*; data reduction: *DENZO* and *COLLECT*; program(s) used to solve structure: *SHELXS97* (Sheldrick, 2008 ▶); program(s) used to refine structure: *SHELXL97* (Sheldrick, 2008 ▶); molecular graphics: *ORTEP-3* (Farrugia, 1997 ▶) and *DIAMOND* (Brandenburg, 2006 ▶); software used to prepare material for publication: *publCIF* (Westrip, 2010 ▶).

## Supplementary Material

Crystal structure: contains datablock(s) global, I. DOI: 10.1107/S1600536811038281/qm2029sup1.cif

Structure factors: contains datablock(s) I. DOI: 10.1107/S1600536811038281/qm2029Isup2.hkl

Additional supplementary materials:  crystallographic information; 3D view; checkCIF report

## Figures and Tables

**Table 1:** Selected bond lengths (Å)

Sn—Cl1	2.3919 (6)
Sn—Cl2	2.4144 (6)
Sn—Cl3	2.4690 (6)
Sn—O1	2.2129 (17)
Sn—O2	2.1850 (18)
Sn—C1	2.135 (2)

**Table 2:** Hydrogen-bond geometry (Å, °)

*D*—H⋯*A*	*D*—H	H⋯*A*	*D*⋯*A*	*D*—H⋯*A*
N1—H1n⋯Cl3^i^	0.81 (3)	2.60 (3)	3.366 (3)	159 (3)
N1—H2n⋯Cl1^ii^	0.78 (3)	2.76 (3)	3.519 (3)	164 (3)
N2—H3n⋯Cl3^iii^	0.90 (3)	2.55 (3)	3.435 (2)	170 (3)
N2—H4n⋯Cl1^iv^	0.89 (3)	2.59 (3)	3.432 (2)	160 (3)
N2—H4n⋯O1^iv^	0.89 (3)	2.66 (3)	3.219 (3)	122 (2)
N3—H5n⋯Cl1^iv^	0.86 (3)	2.85 (3)	3.584 (3)	145 (3)
N3—H5n⋯Cl3^v^	0.86 (3)	2.85 (3)	3.469 (2)	131 (2)
N3—H6n⋯Cl2	0.80 (3)	2.54 (3)	3.272 (3)	152 (3)

Symmetry codes: (i) 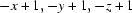; (ii) 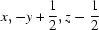; (iii) 
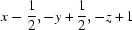
; (iv) 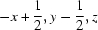; (v) 
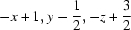
.

**Fig. 1.**
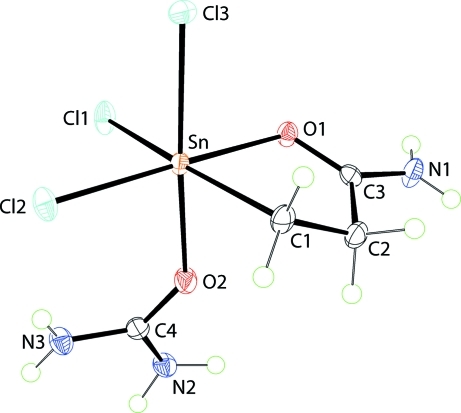
The molecular structure of (I) showing the atom-labelling scheme and displacement ellipsoids at the 50% probability level.

**Fig. 2.**
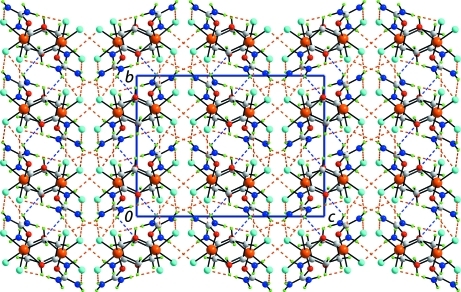
A view in projection down the a axis of the unit-cell contents of (I). The The N—H···O and N—H···Cl hydrogen bonds are shown as blue and orange dashed lines, respectively.

## supplementary crystallographic information

### Comment

The title compound, (I), was investigated as part of a wider study of 2-amidoethyl-tin compounds (Tiekink *et al.*, 2006; Wardell *et al.*, 2010, Howie *et al.*, 2011, and references therein). The title compound was obtained by a ligand exchange reaction between (H_2_NCOCH_2_CH_2_-*C*,*O*)(EtCONH_2_-*O*)SnCl_3_, obtained as previously reported (Howie *et al.*, 2011), and urea.

The Sn atom in (I), Fig. 1, is octahedrally coordinated within a CCl_3_O_2_ provided by the C,*O* donors derived from a chelating amidoethyl ligand, a urea-O atom and three Cl atoms, the latter are arranged facially. There is significant disparity in the Sn—Cl bond distances, Table 1. As observed in the related Sn[OCH(NH_2_)CH_2_CH_2_]Cl_3_L structures where *L* = 3-chloropropionamide (Tiekink *et al.* (2006) and *L* = water (co-crystallized as a 1:2 18-crown-6 complex) (Wardell *et al.*, 2010), these can be explained in terms to the relative *trans* influence exerted by the remaining donor atoms. Thus, the Cl1 atom, *trans* to a C atom, forms a significantly shorter Sn—Cl bond than the other Cl atoms. The elongation of the Sn—Cl3 bond distance with respect to the Sn—O2 distance is related to the stronger coordinating ability of the urea-O atom. Distortions from the ideal octahedral geometry are not great with the maximum deviation found in the C1—Sn—Cl1 angle of 162.84 (7) °.

As anticipated with three amino residues in the structure, there are significant hydrogen bonding interactions operating in the crystal structure of (I), Table 2. While all amino-H participate in hydrogen bonding interactions, Table 2, two of these atoms, *i.e*. H4n and H5n, are bifurcated and so the hydrogen bonding distances are relatively long. The hydrogen bonding scheme leads to a three-dimensional architecture, Fig. 2.

### Experimental

A solution of the complex, (H_2_NCOCH_2_CH_2_-*C*,*O*)(EtCONH_2_-*O*)SnCl_3_, isolated from a reaction mixture containing SnCl_2_, HCl and H_2_C═CHCONH_2_ in Et_2_O (Howie *et al.*, 2011) (0.74 g, 2 mmol) and urea (10 mmol) in ethanol (20 ml) was heated at 313 K for 30 min. Crystals of (H_2_NCOCH_2_CH_2_-*C*,*O*)(H_2_NCONH_2_-*O*)SnCl_3_ (I) were harvested from the reaction solution maintained at room temperature, *M*.pt. 469–471 K. The sample used in the structure determination was grown from its acetone solution. (IR, cm^-1^): 3300–2500 (v. br), 1698 (s, br), 1590, 1574, 1486, 1427, 1411, 1303, 1263, 1139, 1087, 1102, 1087, 1074, 1004, 916, 898, 850, 808, 750, 719, 667, 652, 544, 496, 416.

### Refinement

The C-bound H atoms were geometrically placed (C–H = 0.99 Å) and refined as riding with *U*_iso_(H) = 1.2*U*_eq_(C). The N-bound H atoms were located from a difference map and their positions refined with *U*_iso_(H) = 1.2*U*_eq_(N).

### Crystal data

**Table:**

[Sn(C_3_H_6_NO)Cl_3_(CH_4_N_2_O)]	*F*(000) = 1376
*M**_r_* = 357.19	*D*_x_ = 2.212 Mg m^−^^3^
Orthorhombic, *P**b**c**a*	Mo *K*α radiation, λ = 0.71073 Å
Hall symbol: -P 2ac 2ab	Cell parameters from 2702 reflections
*a* = 11.1223 (2) Å	θ = 2.9–27.5°
*b* = 12.0180 (3) Å	µ = 3.10 mm^−^^1^
*c* = 16.0461 (5) Å	*T* = 120 K
*V* = 2144.85 (9) Å^3^	Plate, colourless
*Z* = 8	0.14 × 0.08 × 0.03 mm

### Data collection

**Table:**

Bruker–Nonius APEXII CCD camera on κ-goniostat diffractometer	2451 independent reflections
Radiation source: Bruker-Nonius FR591 rotating anode	2242 reflections with *I* > 2σ(*I*)
10cm confocal mirrors	*R*_int_ = 0.039
Detector resolution: 9.091 pixels mm^-1^	θ_max_ = 27.5°, θ_min_ = 3.1°
φ & ω scans	*h* = −13→14
Absorption correction: multi-scan (*SADABS*; Sheldrick, 2007)	*k* = −12→15
*T*_min_ = 0.738, *T*_max_ = 0.913	*l* = −19→20
12333 measured reflections	

### Refinement

**Table:**

Refinement on *F*^2^	Primary atom site location: structure-invariant direct methods
Least-squares matrix: full	Secondary atom site location: difference Fourier map
*R*[*F*^2^ > 2σ(*F*^2^)] = 0.022	Hydrogen site location: inferred from neighbouring sites
*wR*(*F*^2^) = 0.051	H atoms treated by a mixture of independent and constrained refinement
*S* = 1.10	*w* = 1/[σ^2^(*F*_o_^2^) + (0.*P*)^2^ + 3.4389*P*] where *P* = (*F*_o_^2^ + 2*F*_c_^2^)/3
2451 reflections	(Δ/σ)_max_ = 0.001
136 parameters	Δρ_max_ = 0.42 e Å^−^^3^
0 restraints	Δρ_min_ = −0.41 e Å^−^^3^

### Special details

**Table:**

Geometry. All s.u.'s (except the s.u. in the dihedral angle between two l.s. planes) are estimated using the full covariance matrix. The cell s.u.'s are taken into account individually in the estimation of s.u.'s in distances, angles and torsion angles; correlations between s.u.'s in cell parameters are only used when they are defined by crystal symmetry. An approximate (isotropic) treatment of cell s.u.'s is used for estimating s.u.'s involving l.s. planes.
Refinement. Refinement of *F*^2^ against ALL reflections. The weighted *R*-factor *wR* and goodness of fit *S* are based on *F*^2^, conventional *R*-factors *R* are based on *F*, with *F* set to zero for negative *F*^2^. The threshold expression of *F*^2^ > 2σ(*F*^2^) is used only for calculating *R*-factors(gt) *etc*. and is not relevant to the choice of reflections for refinement. *R*-factors based on *F*^2^ are statistically about twice as large as those based on *F*, and *R*- factors based on ALL data will be even larger.

### Fractional atomic coordinates and isotropic or equivalent isotropic displacement parameters (Å^2^)

**Table:**

	*x*	*y*	*z*	*U*_iso_*/*U*_eq_	
Sn	0.536685 (14)	0.238549 (14)	0.600556 (10)	0.01156 (6)	
Cl1	0.40499 (6)	0.32834 (5)	0.69733 (4)	0.02118 (14)	
Cl2	0.61521 (5)	0.11894 (5)	0.70850 (4)	0.01937 (14)	
Cl3	0.69075 (6)	0.38320 (5)	0.62367 (4)	0.01866 (14)	
O1	0.44877 (15)	0.34588 (14)	0.50652 (10)	0.0147 (4)	
O2	0.38496 (16)	0.12814 (16)	0.57517 (11)	0.0202 (4)	
N1	0.3982 (2)	0.3491 (2)	0.37174 (14)	0.0194 (5)	
H1N	0.360 (3)	0.405 (3)	0.378 (2)	0.023*	
H2N	0.403 (3)	0.321 (3)	0.328 (2)	0.023*	
N2	0.2038 (2)	0.0487 (2)	0.58401 (15)	0.0177 (5)	
H3N	0.191 (3)	0.069 (2)	0.531 (2)	0.021*	
H4N	0.160 (3)	−0.006 (3)	0.6044 (18)	0.021*	
N3	0.3448 (2)	0.01609 (19)	0.68601 (14)	0.0182 (5)	
H5N	0.296 (3)	−0.026 (2)	0.713 (2)	0.022*	
H6N	0.411 (3)	0.024 (3)	0.7044 (19)	0.022*	
C1	0.6109 (2)	0.1691 (2)	0.48944 (15)	0.0166 (5)	
H1A	0.6918	0.2004	0.4789	0.020*	
H1B	0.6183	0.0873	0.4949	0.020*	
C2	0.5261 (2)	0.1983 (2)	0.41798 (16)	0.0173 (5)	
H2A	0.5736	0.2077	0.3663	0.021*	
H2B	0.4694	0.1359	0.4092	0.021*	
C3	0.4551 (2)	0.3040 (2)	0.43435 (15)	0.0141 (5)	
C4	0.3135 (2)	0.0653 (2)	0.61521 (15)	0.0148 (5)	

### Atomic displacement parameters (Å^2^)

**Table:**

	*U*^11^	*U*^22^	*U*^33^	*U*^12^	*U*^13^	*U*^23^
Sn	0.01312 (11)	0.01191 (10)	0.00966 (10)	0.00196 (6)	0.00039 (6)	0.00036 (6)
Cl1	0.0263 (3)	0.0240 (3)	0.0133 (3)	0.0119 (3)	0.0043 (3)	0.0018 (2)
Cl2	0.0177 (3)	0.0225 (3)	0.0179 (3)	0.0042 (2)	−0.0017 (2)	0.0074 (2)
Cl3	0.0221 (3)	0.0154 (3)	0.0185 (3)	−0.0029 (2)	−0.0031 (2)	−0.0013 (2)
O1	0.0177 (9)	0.0146 (9)	0.0119 (8)	0.0039 (7)	0.0003 (7)	0.0000 (7)
O2	0.0207 (9)	0.0254 (10)	0.0145 (9)	−0.0095 (8)	−0.0003 (7)	0.0025 (7)
N1	0.0258 (12)	0.0216 (13)	0.0107 (10)	0.0070 (10)	−0.0013 (9)	−0.0022 (9)
N2	0.0149 (11)	0.0195 (12)	0.0187 (11)	−0.0038 (9)	0.0006 (9)	0.0023 (9)
N3	0.0162 (11)	0.0210 (12)	0.0175 (11)	−0.0018 (9)	0.0016 (9)	0.0041 (9)
C1	0.0182 (12)	0.0183 (13)	0.0132 (12)	0.0035 (10)	0.0031 (10)	−0.0027 (9)
C2	0.0227 (13)	0.0172 (13)	0.0120 (11)	0.0022 (11)	0.0008 (10)	−0.0027 (10)
C3	0.0121 (11)	0.0155 (13)	0.0148 (12)	−0.0042 (9)	0.0029 (9)	0.0018 (10)
C4	0.0170 (12)	0.0125 (12)	0.0148 (12)	0.0033 (10)	0.0032 (9)	−0.0018 (9)

### Geometric parameters (Å, °)

**Table:**

Sn—Cl1	2.3919 (6)	N2—H3N	0.90 (3)
Sn—Cl2	2.4144 (6)	N2—H4N	0.89 (3)
Sn—Cl3	2.4690 (6)	N3—C4	1.327 (3)
Sn—O1	2.2129 (17)	N3—H5N	0.86 (3)
Sn—O2	2.1850 (18)	N3—H6N	0.80 (3)
Sn—C1	2.135 (2)	C1—C2	1.526 (3)
O1—C3	1.265 (3)	C1—H1A	0.9900
O2—C4	1.271 (3)	C1—H1B	0.9900
N1—C3	1.305 (3)	C2—C3	1.519 (4)
N1—H1N	0.81 (3)	C2—H2A	0.9900
N1—H2N	0.78 (3)	C2—H2B	0.9900
N2—C4	1.334 (3)		
			
C1—Sn—O2	84.59 (9)	H3N—N2—H4N	117 (3)
C1—Sn—O1	80.18 (8)	C4—N3—H5N	121 (2)
O2—Sn—O1	83.43 (7)	C4—N3—H6N	120 (2)
C1—Sn—Cl1	162.84 (7)	H5N—N3—H6N	118 (3)
O2—Sn—Cl1	85.53 (5)	C2—C1—Sn	107.38 (16)
O1—Sn—Cl1	84.79 (5)	C2—C1—H1A	110.2
C1—Sn—Cl2	103.09 (7)	Sn—C1—H1A	110.2
O2—Sn—Cl2	92.95 (5)	C2—C1—H1B	110.2
O1—Sn—Cl2	174.92 (5)	Sn—C1—H1B	110.2
Cl1—Sn—Cl2	91.39 (2)	H1A—C1—H1B	108.5
C1—Sn—Cl3	97.60 (7)	C3—C2—C1	112.6 (2)
O2—Sn—Cl3	172.57 (5)	C3—C2—H2A	109.1
O1—Sn—Cl3	89.92 (5)	C1—C2—H2A	109.1
Cl1—Sn—Cl3	90.56 (2)	C3—C2—H2B	109.1
Cl2—Sn—Cl3	93.46 (2)	C1—C2—H2B	109.1
C3—O1—Sn	111.56 (15)	H2A—C2—H2B	107.8
C4—O2—Sn	138.58 (16)	O1—C3—N1	120.8 (2)
C3—N1—H1N	121 (2)	O1—C3—C2	121.4 (2)
C3—N1—H2N	119 (2)	N1—C3—C2	117.8 (2)
H1N—N1—H2N	120 (3)	O2—C4—N3	122.2 (2)
C4—N2—H3N	117.7 (19)	O2—C4—N2	118.1 (2)
C4—N2—H4N	119 (2)	N3—C4—N2	119.7 (2)
			
C1—Sn—O1—C3	16.38 (17)	O1—Sn—C1—C2	−22.51 (17)
O2—Sn—O1—C3	−69.23 (16)	Cl1—Sn—C1—C2	6.7 (4)
Cl1—Sn—O1—C3	−155.32 (16)	Cl2—Sn—C1—C2	153.52 (16)
Cl2—Sn—O1—C3	−114.0 (5)	Cl3—Sn—C1—C2	−111.11 (17)
Cl3—Sn—O1—C3	114.11 (15)	Sn—C1—C2—C3	26.7 (3)
C1—Sn—O2—C4	139.7 (3)	Sn—O1—C3—N1	173.13 (19)
O1—Sn—O2—C4	−139.6 (3)	Sn—O1—C3—C2	−5.3 (3)
Cl1—Sn—O2—C4	−54.3 (3)	C1—C2—C3—O1	−14.9 (3)
Cl2—Sn—O2—C4	36.8 (3)	C1—C2—C3—N1	166.6 (2)
Cl3—Sn—O2—C4	−112.8 (4)	Sn—O2—C4—N3	−28.6 (4)
O2—Sn—C1—C2	61.74 (17)	Sn—O2—C4—N2	151.7 (2)

### Hydrogen-bond geometry (Å, °)

**Table:**

*D*—H···*A*	*D*—H	H···*A*	*D*···*A*	*D*—H···*A*
N1—H1n···Cl3^i^	0.81 (3)	2.60 (3)	3.366 (3)	159 (3)
N1—H2n···Cl1^ii^	0.78 (3)	2.76 (3)	3.519 (3)	164 (3)
N2—H3n···Cl3^iii^	0.90 (3)	2.55 (3)	3.435 (2)	170 (3)
N2—H4n···Cl1^iv^	0.89 (3)	2.59 (3)	3.432 (2)	160 (3)
N2—H4n···O1^iv^	0.89 (3)	2.66 (3)	3.219 (3)	122 (2)
N3—H5n···Cl1^iv^	0.86 (3)	2.85 (3)	3.584 (3)	145 (3)
N3—H5n···Cl3^v^	0.86 (3)	2.85 (3)	3.469 (2)	131 (2)
N3—H6n···Cl2	0.80 (3)	2.54 (3)	3.272 (3)	152 (3)

Symmetry codes: (i) −*x*+1, −*y*+1, −*z*+1; (ii) *x*, −*y*+1/2, *z*−1/2; (iii) *x*−1/2, −*y*+1/2, −*z*+1; (iv) −*x*+1/2, *y*−1/2, *z*; (v) −*x*+1, *y*−1/2, −*z*+3/2.
